# The Effects of Hurricane Helene on Mental Health and Physical Activity

**DOI:** 10.13023/jah.0801.02

**Published:** 2026-04-01

**Authors:** Hunter Smathers, Robyn York, Jarrett Walbolt

**Affiliations:** Montreat College; Montreat College; Montreat College

**Keywords:** Appalachia, Natural Disaster, Post-Traumatic Stress Disorder

## Abstract

**Introduction:**

Hurricane Helene brought widespread damage to Southern Appalachia, disrupting utilities, health care, and recreation. While natural disasters are linked to declines in mental and physical health, few studies have examined their effects on distinct types of physical activity (PA).

**Purpose:**

This study evaluated the effects of Hurricane Helene on perceived stress, depressive symptoms, post-traumatic stress disorder (PTSD) symptomology, and intentional (exercise for health benefits) and incidental (physical labor or transportation) PA. Evacuation status influence was also explored.

**Methods:**

Twenty participants completed assessments four weeks before (BASE), immediately after (0POST), and four weeks after landfall (4POST). Measures included the Perceived Stress Scale (PSS), Patient Health Questionnaire-9 (PHQ-9), and the Impact of Event Scale–Revised (IES-R). PA was assessed via activity recalls.

**Results:**

PSS and PHQ-9 increased from BASE to 4POST (*p* = 0.002, *p* = 0.017; respectively). Overall PA levels remained stable, but incidental PA increased at 0POST among non-evacuated individuals (*p* = 0.002). Time evacuated negatively predicted hyperarousal (*p* = 0.007) and PSS change (*p* = 0.006). Baseline stress positively predicted intentional exercise at 0POST (*p* = 0.008), while higher PQH-9 at 4POST predicted reduced intentional exercise (*p* = 0.038).

**Implications:**

Hurricane Helene increased psychological distress in a rural Appalachian population. While incidental PA increased immediately post-Helene in non-evacuated individuals, it may have exposed them to ongoing stressors. Evacuation status influenced mental health outcomes, suggesting non-evacuated individuals may face greater psychological risks. Recovery efforts in Appalachian communities should prioritize mental health support, promote exercise behaviors, and ensure infrastructure for safe evacuation.

## INTRODUCTION

Hurricane Helene was a category 4 hurricane that brought significant flooding and wind damage to the Southeastern United States (U.S.), including Western North Carolina (WNC). The resulting damage devastated local communities and resulted in greatly impaired access to fitness resources, parks, trail systems, and infrastructure. Further, tens of thousands of homes sustained damage. With around 4.6 million Carolinians living in affected counties, countless individuals lost access to water, electricity, and healthcare facilities.^1^ An estimated $59.6 billion in damage occurred in North Carolina (NC) alone.

Disasters, both man-made and natural, can affect human health at both the individual and population levels. A review of the literature indicates these disaster conditions are linked to altered physical, mental, and social health, as well as limited access to health care and changes to environmental risk.^2^ Following Hurricane Sandy, youth living within the affected region had significant deterioration in health behaviors.^3^ While exposure to impacted areas was not associated with negative changes in mental health outcomes, there were significant differences in physical activity (PA), sexual activity, and substance abuse.^3^ Among 392 low-income individuals exposed to Hurricane Katrina, there were significant negative mental and physical health outcomes persisting a year later.^4^ While post-traumatic stress disorder (PTSD) symptomology was not assessed prior to the disaster, an Impact of Event Scale - Revised (IES-R) given post-Katrina found 47.7% of the participants to be classified as having probable PTSD.^4^

Previous research has indicated that one’s physical environment can affect their PA levels.^5^ Additionally, PA is negatively altered because of climate change associated with natural disasters, air pollution, and carbon footprint.^6^ Daily bicycle and pedestrian counts following Hurricane Harvey declined immediately following the Hurricane but returned to normal levels after six weeks.^7^ These changes in activity did not account for infrastructure damage or environmental determinants which may have affected the counts. Access to fitness equipment and instruction is critical to keeping individuals engaged in PA, particularly in disabled populations.^8^,^9^ During the Covid-19 pandemic, changes in access to fitness resources were a primary barrier to maintenance of PA.^10^ Following the Great East Japan Earthquake, it was found that one’s access to fitness equipment played a role in the likelihood of one’s engagement in PA.^5^ In a study conducted following a firework explosion in the Netherlands, it was found that survivors who experienced a damaged house, losing a relative or friend, or experiencing a physical injury or severe anxiety because of the disaster were more likely to be challenged with physical and mental health problems.^11^

The aim of this study was to examine the effects of Hurricane Helene on mental health and PA. Previous research has sought to learn the effects of natural disasters on PA but has not closely examined the distinction between intentional and incidental exercise. With that in mind, the study aimed to examine the differences between intentional and incidental exercise following Helene. Additionally, we sought to determine if higher engagement in PA or any hurricane related experiences had any relationship with participants’ stress scores and mental health issues resulting from the natural disaster.

## METHODS

### Data Collection and Participants

Data collection was completed for three timepoints:

1. Baseline (BASE): Occurred four weeks prior to Hurricane Helene’s landfall,
2. Immediately Post-Helene (0POST): Occurred in the week following Hurricane Helene’s landfall,
3. Four Weeks Post-Helene (4POST): Occurred four weeks after Hurricane Helene’s landfall.

Participants of this study were initially part of a study that was interrupted by Hurricane Helene assessing the effectiveness of an outdoor walking group. BASE data was collected for 26 initial participants. Out of those 26, 20 were successfully contacted after Hurricane Helene for further data collection. Table 1 presents demographic data for the participants. To be eligible for the study, participants were required to be 18 years of age and have no contraindications to exercise. Participants had completed four weeks in a walking group prior to Hurricane Helene making landfall. Both the original study and the study modifications following Hurricane Helene were approved by an institutional review board.

### Measures

#### Perceived Stress

The Perceived Stress Scale (PSS) was administered at BASE and 4POST to assess participants’ perceived levels of stress.^12^ The PSS is a widely used self-report questionnaire designed to measure the degree to which situations in the participant’s life are perceived as stressful. The 10-item version of the scale was used in the present study. Participants were instructed to rate how often they experienced certain thoughts and feelings related to stress during the past month. Items were rated on a 5-point Likert scale ranging from 0 (Never) to 4 (Very often). Scores were summed to produce a total score, with higher scores indicating greater perceived stress.

#### Depression Symptoms

The Patient Health Questionnaire-9 (PHQ-9) was administered at BASE and 4POST to assess symptoms of depression.^13^ The PHQ-9 is a nine-item self-report questionnaire. Participants were instructed to rate how often they experienced each depressive symptom over the previous two weeks. The scale was a 4-point Likert scale ranging from 0 (Not at all) to 3 (Nearly every day). The PHQ-9 provides a total score ranging from 0 to 27, with higher scores indicating greater severity of depressive symptoms.

#### PTSD Symptoms

The Impact of Event Scale - Revised (IES-R) was given at 4POST to assess potential symptoms of PTSD.^14^ The IES-R is a 22-item self-report questionnaire. Participants were instructed to identify how often they experienced the listed hurricane-related symptoms in the previous week. The scale was a 5-point scale ranging from 0 (Not at all) to 4 (Extremely). The IES-R features sub-scales for the symptom clusters of hyperarousal, intrusion, and avoidance, along with a total score. Total scores of 25 or above were used to designate high IES-R classification, while below 25 was used to designate low IES-R classification.^15^

#### Physical Activity

Seven-Day Activity Recalls were used to measure PA at two time points.^16^ At 0POST, the Seven-Day Activity Recall was used to measure exercise during the week immediately following the storm. Participants again completed a Seven-Day Activity Recall at 4POST. The dates represented by the measure completed at 0POST were September 28 – October 4, 2024. The dates represented by the measure done 4POST were October 26 – November 1, 2024. The Seven-Day Activity Recall questionnaire assessed moderate, hard, and very hard activities in which participants were involved. Participants were not surveyed on their participation in light activities. For this study, light activities included standing, driving, and walking leisurely. Additionally, the survey asked participants about their activity levels during the weekdays (Monday–Friday) and for the weekends (Saturday–Sunday).

At both times when participants completed the Seven-Day Activity Recall, there were two questionnaires they completed. One of the questionnaires was based on activity that was “incidental exercise,” and the other survey was based on PA that was “intentional exercise.” For this study, intentional exercise was defined as “exercise performed for the sake of exercise, and the physical and mental benefits of exercise.” Conversely, incidental exercise was defined as “exercise performed as part of one’s occupation, transportation, chores, or recovery efforts, but not specifically for the benefits of exercise.” These definitions were defined for participants in the survey directions.

#### Hurricane Related Experiences

Participants were asked how many days they were in an area affected by Hurricane Helene. While all participants were in an area affected by Hurricane Helene as it struck WNC, many participants evacuated in the days or weeks after. If a participant left their initial location but remained in an affected area, this was not considered an evacuation for the purposes of this study.

### Statistical Analyses

Repeated measures analyses of variances (ANOVAs) were used to evaluate changes in PSS, PHQ-9, and exercise across timepoints and with consideration to the group factors of whether or not the participant evacuated and IES-R classification. The Shapiro-Wilk and Levene tests were used to verify the assumption of normality and homogeneity of variances. Bivariate correlations were used initially to identify correlated variables for further analysis. These significant values were further investigated with simple linear regressions run individually to identify relationships between variables. Eta-squared metrics (η^2^_p_) were used to assess effect size with 0.01 representing small, 0.06 representing medium, and 0.14 representing large effects.^17^ All statistical analyses were performed using SPSS 29 (IBM Corporation, Armonk, NY, USA). Statistical significance was set at *p* ≤ 0.05.

## RESULTS

PTSD Symptomology population characteristics are shown in Table 2.

The main effect of time was found to be significant for PSS scores (F (1,19) = 11.2, *p* = 0.003, η^2^_p_ = 0.371). PSS scores from BASE to 4POST rose from 13.7 ± 1.77 to 20.2 ± 1.46 (*p* = 0.002). No group and time interaction was found for the groups of IES-R classification (F (1,18) = 1.21, *p* = 0.285, η^2^_p_ = 0.063) or whether they evacuated (F (1,16) = 3.24, *p* = 0.091, η^2^_p_ = 0.169) for PSS.

The main effect of time was found to be significant for PHQ-9 scores (F (1,19) = 6.85, *p* = 0.017, η^2^_p_ = 0.265). PHQ-9 scores rose from 3.44 ± 0.74 to 6.16 ± 0.89. An interaction between IES-R classification and time was found for PHQ-9 scores (F (1,18) = 7.62, *p* = 0.013, η^2^_p_ = 0.297). From BASE to 4POST, PHQ-9 scores for the high IES-R classification rose significantly from 3.22 ± 0.76 to 8.33 ± 1.07 (*p* < 0.001), while the low IES-R classification had no significant change (*p* = 0.693). No interaction was found between whether they evacuated and time for PHQ-9 (F (1,16) = 0.714, *p* = 0.411, η^2^_p_ = 0.043).

The main effect of time was not significant for incidental exercise (F (1,15) = 0.087, *p* = 0.773, η^2^_p_ = 0.006), intentional exercise (F (1,15) = 0.758, *p* = 0.398, η^2^_p_ = 0.048), or total exercise (F (1,15) = 0.139, *p* = 0.715, η^2^_p_ = 0.009). No IES-R category group and time interactions were found for incidental exercise (F (1,14) = 0.034, *p* = 0.857, η^2^_p_ = 0.002), intentional exercise (F (1,14) = 0.181, *p* = 0.677, η^2^_p_ = 0.013), or total exercise (F (1,15) = 0.022, *p* = 0.884, η^2^_p_ = 0.002). A significant interaction effect was found between whether they evacuated and time for incidental exercise (F (1,14) = 8.23, *p* = 0.012, η^2^_p_ = 0.370). At 0POST, those who did not evacuate had engaged in significantly more incidental exercise than those who did evacuate (26.7 ± 21.5 vs. 5.31 ± 3.94 hours per week, *p* = 0.002). Additionally, in those who did not evacuate, incidental exercise from 0POST to 4POST decreased from 26.7 ± 21.5 to 9.00 ± 1.80 hours per week (*p* = 0.016). No significant interaction effect was found between whether they evacuated and time for intentional exercise (F (1,14) = 0.225, *p* = 0.643, η^2^_p_ = 0.016) or total exercise (F (1,14) = 2.49, *p* = 0.137, η^2^_p_ = 0.151).

Bivariate correlation found a significant negative correlation between hyperarousal and days spent evacuated (r = −0.586, *p* = 0.007). Despite this correlation, days spent evacuated were not correlated with avoidance (r = 0.200, *p* = 0.200), intrusion (r = −0.032, *p* = 0.451), or the total IES-R score (r = −0.120, *p* = 0.323). Days spent evacuated had a significant negative correlation with change in PSS scores (r = −0.598, *p* = 0.006).

A significant positive correlation was found between PSS and PHQ-9 at BASE (r = 0.573, *p* = 0.004). At 4POST, PSS and PHQ-9 were again significantly correlated (r = 0.753, *p* < 0.001). Both PSS and PHQ-9 were significantly positively correlated with hyperarousal at 4POST (r = 0.560, *p* = 0.005, and r = 0.607, *p* = 0.002; respectively). At 4POST, both PSS and PHQ-9 were also significantly positively correlated with IES-R total (r = 0.452, *p* = 0.023, and r = 0.540, *p* = 0.007; respectively). PSS at BASE was significantly positively correlated with intentional exercise at 0POST (r = 0.527, *p* = 0.010). PHQ-9 at 4POST was significantly negatively correlated with intentional exercise at 4POST (r = −0.506, *p* = 0.019).

Simple linear regressions were run individually to evaluate relationships between variables related to exercise, IES-R scores, PHQ-9, PSS, and factors related to evacuation. The results of the regression found that days spent evacuated explained 34.3% of the variance in hyperarousal (F (1,15) = 7.84, *p* = 0.013). Days spent evacuated explained 35.8% of the variance in change in PSS scores (F (1,15) = 8.36, *p* = 0.011).

PSS at BASE explained 32.8% of the variance in PHQ-9 at BASE (F (1,18) = 8.79, *p* = 0.008). At 4POST, PSS explained 56.6% of the variance in PHQ-9 (F (1,18) = 23.5, *p* < 0.001). At 4POST, independently run regression analyses showed that PSS and PHQ-9 explained 31.4% and 36.9% of variance, respectively, in hyperarousal (F (1,18) = 8.23, *p* = 0.010, and F(1,18) = 10.5, *p* = 0.005; respectively). Additionally, at 4POST, independently run regression analyses showed that PSS and PHQ-9 explained 20.4% and 29.1% of variance, respectively, in IES-R total (F (1,18) = 4.63, *p* = 0.045, and F(1,18) = 7.39, *p* = 0.014; respectively). PSS at BASE explained 27.8% of the variance in intentional exercise at 0POST (F (1,17) = 6.55, *p* = 0.020). At 4POST, PHQ-9 explained 25.6% of variance in intentional exercise (F (1,15) = 5.17, *p* = 0.038).

## DISCUSSION

Perceived stress and depressive symptoms increased significantly after Hurricane Helene. For those with a high IES-R classification, depressive symptoms were found to increase even more dramatically. Psychological states such as stress, depression, and PTSD have a high degree of comorbidity, explaining the links found between them.^18^

Those who didn’t evacuate completed more incidental exercise immediately after Hurricane Helene. This was likely driven by those who remained in an affected area engaging in cleanup efforts and exercising as a means of travel due to infrastructure damage. Incidental exercise has been shown previously to improve health.^19^ While this may seem healthy initially, incidental exercise has its downsides in the context of disaster. Individuals engaging in incidental exercise within a disaster affected region may be inadvertently increasing their exposure to traumatic events.^20^ Additionally, the work itself may be mentally stressful, thus counteracting the normal anxiolytic effect of exercise.^21^

While no significant time-based changes were found for any kind of exercise, participants anecdotally reported a wide array of changes, with some decreasing exercise and others increasing exercise, but very few keeping the same exercise patterns. Perceived stress prior to the hurricane predicted increased intentional exercise immediately after. Decreased intentional exercise predicted higher depressive symptoms a month after the storms. This supports the idea that exercise habits diverge based on whether exercise is used as a coping mechanism or is limited due to depressive symptoms.

Likely due to lower exposure to traumatic events, those who evacuated had significantly different psychological and exercise responses. Hyperarousal, in particular, was found to be linked to the number of days an individual spent evacuated. This may mean that individuals who are more attached to a region, own a home, or have financial or health limitations to their ability to evacuate may require improved strategies to maintain their mental and physical health following a disaster. Other studies have suggested that education, access to physical resources, and infrastructure is critical to Appalachian communities.^22^

In combination due to design and inability to foresee natural disasters, this study has several limitations. Participants unable to be contacted to complete follow-up exercise and psychological questionnaires after Hurricane Helene may skew the sample. These individuals may have had more severe psychopathology due to greater hurricane-related impacts or may have undergone a more extensive evacuation. Due to the initial study design, prior to modification due to Hurricane Helene, our study is limited by the use of self-report metrics. The IES-R was used in this study despite the PTSD Checklist for DSM-5 (PCL-5) assessing all domains of the current PTSD criteria. Despite this, the IES-R has been found to have concurrent validity with the PCL-5.^23^ This was further complicated by ongoing severe communication limitations throughout the post-hurricane study period.

This study would have also been improved by having some baseline information that had not been foreseen being relevant prior to the hurricane. Additionally, due to the unique circumstances surrounding this study, a priori power analysis was not possible. The study was limited to participants enrolled in a previous study; thus, the sample size may be too small to detect some effects. Despite these limitations, this study is unique in having some baseline mental health data to help establish causal links.

## CONCLUSION

Hurricane Helene led to worsened psychological health in individuals within a rural community of WNC. Exercise patterns shifted within the month following the storm, providing evidence for a conflicting effect of intentional exercise potentially being used as a coping mechanism for stress, but decreasing in individuals with higher depressive symptoms. Individuals who did not evacuate had higher incidental exercise immediately after the hurricane than those who did. These same individuals who did not evacuate also are likely at higher risk of worsened psychological health. Policymakers and future researchers should consider interventions related to education and improved access to exercise, mental health, and infrastructure resources during and after natural disasters in Appalachia.

**SUMMARY BOX**

**What is already known about this topic?**

Natural disasters, including hurricanes, disrupt normal physical activity and daily living routines. Exposure to trauma such as damaged infrastructure, supply and food shortages, extreme weather, and harm to human life results in worsened mental health. Maintaining mental and physical health during natural disasters is a challenge facing Appalachian communities in the future.

**What is added by this report?**

Exposure to hurricane-related trauma likely resulted in worsened mental health and disturbed exercise patterns. Individuals who didn’t evacuate engaged in significant incidental exercise, likely due to aiding in recovery efforts. This may have also meant greater exposure to trauma and worsened psychological states.

**What are the implications for future research?**

Future research should investigate methods to prevent worsened psychological symptoms following natural disasters. Identifying ways to promote sustainable healthy exercise patterns in the aftermath are of particular note.

## Figures and Tables

**Table 1:** Baseline Participant Characteristics

Factor	Mean Values ± SD		
	Male	Female	Total
n	5	15	20
Age (years)	22.6 ± 6.95	30.2 ± 14.9	28.3 ± 13.6
Body Fat (%)	11.4 ± 6.76	27.4 ± 8.79	23.4 ± 10.8
PSS	14.6 ± 2.97	13.3 ± 6.09	13.7 ± 5.43
PHQ-9	5.40 ± 1.82	2.80 ± 2.01	3.45 ± 2.24

**Table 2:** IES-R Subscales and Total

Sub-Score	Mean Values ± SD
IES-R Total	22.6 ± 10.6
IES-R Intrusion	11.0 ± 5.51
IES-R Avoidance	7.55 ± 4.50
IES-R Hyperarousal	4.05 ± 3.41
